# Supplementary material for: Noise-induced plasticity of KCNQ2/3 and HCN channels underlies vulnerability and resilience to tinnitus
Source: eLife. 2015 Aug 27;4:e07242. doi: 10.7554/eLife.07242 (PMC4592936; doi:10.7554/eLife.07242)
Supplement: Supplementary file 1. — Spike parameters of fusiform cells from control and non-tinnitus mice. Spike threshold: control, n = 8, non-tinnitus, n = 8, p = 0.16; spike amplitude: control, n = 8, non-tinnitus, n = 8, p = 0.98; depolarization slope: control, n = 8, tinnitus, n = 8, p = 0.97; hyperpolarization slope: control, n = 8, tinnitus, n = 8, p = 0.07; half height width: control, n = 8, tinnitus, n = 8, p = 0.09; fast afterhyperpolarization (fAHP): control, n = 8, tinnitus, n = 8, p = 0.68). DOI: http://dx.doi.org/10.7554/eLife.07242.015 [file elife07242s001.pdf]

Table 1. Spike parameters of fusiform cells from control and non-tinnitus mice

	Spike threshold	Spike amplitude	Depolarization slope	Hyperpolarization slope	Half height width	fAHP
Control	-47.1 ± 0.5 mV	40.6 ± 1.2 mV	219.2 ± 7.5 V/s	-165.3 ± 5.5 V/s	0.31 ± 0.01 ms	22.0 ± 1.3 mV
Non-tinnitus	-48.4 ± 0.7 mV	40.5 ± 1.6 mV	206.0 ± 7.6 V/s	-152.0 ± 3.7 V/s	0.33 ± 0.01 ms	22.7 ± 0.8 mV

fAHP: fast afterhyperpolarization
Depolarization slope: maximum depolarizing slope
Hyperpolarization slope: minimum hyperpolarizing slope
